# Regulating the Emission Spectrum of CsPbBr_3_ from Green to Blue via Controlling the Temperature and Velocity of Microchannel Reactor

**DOI:** 10.3390/ma11030371

**Published:** 2018-03-02

**Authors:** Yong Tang, Hanguang Lu, Longshi Rao, Zongtao Li, Xinrui Ding, Caiman Yan, Binhai Yu

**Affiliations:** 1 Engineering Research Centre of Green Manufacturing for Energy-Saving and New Energy Technology, School of Mechanical and Automotive Engineering, South China University of Technology, Guangzhou 510640, China; ytang@scut.edu.cn (Y.T.); guyuexuan1999@163.com (H.L.); memerls@mail.scut.edu.cn (L.R.); chamenyan@163.com (C.Y.); mebhaiyu@scut.edu.cn (B.Y.); 2 Department of Mechanical Engineering, University of California, Berkeley, CA 94720, USA; ding.xinrui@mail.scut.edu.cn

**Keywords:** temperature control, velocity control, inorganic perovskite quantum dots, wide spectrum, microchannel reactor

## Abstract

The ability to precisely obtain tunable spectrum of lead halide perovskite quantum dots (QDs) is very important for applications, such as in lighting and display. Herein, we report a microchannel reactor method for synthesis of CsPbBr_3_ QDs with tunable spectrum. By adjusting the temperature and velocity of the microchannel reactor, the emission peaks of CsPbBr_3_ QDs ranging from 520 nm to 430 nm were obtained, which is wider than that of QDs obtained in a traditional flask without changing halide component. The mechanism of photoluminescence (PL) spectral shift of CsPbBr_3_ QDs was investigated, the result shows that the supersaturation control enabled by the superior mass and heat transfer performance in the microchannel is the key to achieve the wide range of PL spectrum, with only a change in the setting of the temperature controller required. The wide spectrum of CsPbBr_3_ QDs can be applied to light-emitting diodes (LEDs), photoelectric sensors, lasers, etc.

## 1. Introduction

Lead halide perovskite quantum dots (QDs) have attracted extensive attention, with outstanding prospects for usage in light-emitting diodes (LEDs), photoelectric sensors, solar cells, and lasers [1,2,3,4,5,6]. As an all inorganic lead halide perovskite quantum dot, CsPbX_3_ (X = Cl, Br, I) has become a promising fluorescence material because of its low cost, narrow emission full width at half maximum (FWHM) (12–42 nm), high luminescence efficiency (90%), stable photochemical property, and simplicity of modification. Moreover, the CsPbX_3_ QDs exhibit broad wavelength coverage (400–700 nm), which is tuned by adjusting the halide ion composition and size scale. The capacity to precisely achieve tunable spectrum and high quality for CsPbX_3_ QDs is extremely significant for application, including in lighting and displays.

Currently, the high-temperature hot-injection method (HI method) and the supersaturated recrystallization method (SR method), are two of the most popular synthetic processes for CsPbX_3_ QDs. For HI method, PbX_2_ (X = Cl, Br, I) precursor solution is fabricated in a high-temperature and inert atmosphere. Next, an octadecene (ODE) solution of cesium compound is injected into the precursor solution, and CsPbX_3_ QDs are subsequently created after cooling to room temperature in a water bath [7]. In 2015, Kovalenko and colleagues synthesized a solution of CsPbX_3_ QDs with cesium carbonate (CsCO_3_), ODE, oleic acid (OA), and halogen lead compounds (PbX_2_) using the HI method [8]. The PL emission of CsPbX_3_ QDs were tuned over the entire visible spectral region of 410–700 nm via the compositional modulation and quantum size effect. In the same year, they found that anion exchange existed in CsPbX_3_ QDs, and the wavelength was also tuned ranging from near ultraviolet to near-infrared, by adjusting the halide ratios in the colloidal nanocrystal solution [9]. To avoid the inconvenient inert gas protection in the HI method, Haibo Zeng and co-workers developed a SR method to synthesize CsPbX_3_ QDs without inert gas production and high temperature [10]. Using this method, the quantum yields (QYs) of 80%, 95%, and 70% for red, green, and blue photoluminescence (PL) spectrum of CsPbX_3_ QDs were obtained, and 90% retention rate after aging 30 d in ambient conditions. In these two methods, the change of PL emission spectrum depended on adjusting the halide components [11,12,13,14].

However, for the above two synthesis methods, turning the PL emission of CsPbX_3_ QDs through changing halide component or ion exchange in the traditional flask is complex, tedious, and time-consuming [15,16,17]. In addition, it is difficult to accurately control the reaction temperature by using flask, which seriously influence the PL emission of the QDs during the synthetic process. Besides, the size of container and the amount of injected solution also influence the reaction temperature. So it is hard to tune the emission spectrum by temperature via regular methods [10]. Therefore, it is essential to develop a powerful and simple tool for the rapid synthesis of CsPbX_3_ QDs with tunable spectrum. Since 1990, when N. Graber et al. proposed the concept of total analysis systems (TAS), microchannel reactors, as the core of microfluidic control technology, have been applied widely in laboratories and the chemical industry because they provide better heat and mass transfer efficiency, a controllable reaction time, facile scaling-up, and superior characteristics compared to the traditional flask [18,19,20].

Here, we demonstrate a microchannel reactor for synthesizing CsPbBr_3_ QDs with tunable spectrum. The PL emission of CsPbBr_3_ QDs can be promptly regulated by changing the process parameters, such as the reaction temperature and the velocity of the precursor solution. By optimizing the synthesis conditions, the emission peak of CsPbBr_3_ QDs can range from green to blue. Moreover, X-ray diffractometer (XRD), transmission electron microscope (TEM), and X-ray photoelectron spectroscopy (XPS) were carried out to characterize the crystal structure, surface morphology, and surface chemical element and valence states. The mechanism of PL spectrum shift was investigated by crystallization theory. The as-prepared CsPbBr_3_ QDs were applied with a red phosphor on a blue LED chip, achieving a high color gamut of 112% National Television Systems Committee (NTSC), which suggested the promise of CsPbBr_3_ QDs for potential applications such as lighting and display.

## 2. Materials and Methods

### 2.1. Materials and Chemicals

To synthesize CsPbBr_3_ QDs, cesium bromide (CsBr, 99.5%), lead bromide (PbBr_2_, 99%), oleic acid (OA, ≥99.0%), oleylamine (OAm, 80–90%), *N*-*N* dimethylformamide (DMF, 99%), chloroform (98%), and toluene (99%) were purchased from Macklin without further purification, are used for the following experiments.

### 2.2. Synthesis of CsPbBr_3_ QDs with Tunable Spectrum

**Figure 1:**
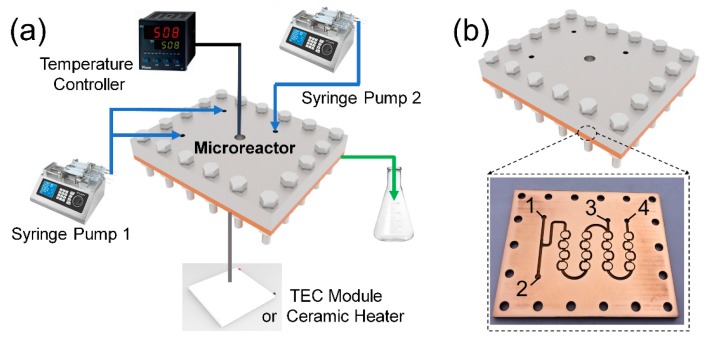
(**a**) Schematic diagram of the microchannel reactor and (**b**) enlarged diagram of the dislocated snake-like microchannel.

To realize effective mixture without mechanical stirring, intense collisions between different fluids are obviously necessary [21,22]. Combining a serpentine channel, a t-type channel, and the dean vortex generated in an arc area, we designed the dislocated snake-like microchannels based on the concept of unbalanced splits and cross-collisions of fluid streams [23,24,25]. For adjustment of the reaction parameters, the microreactor is equipped with syringe pumps and a temperature controller (Figure 1a). A thermal electric cooler module (TEC module) or ceramic heater is attached to the undersurface of the microreactor tightly. Furthermore, the dislocated snake-like microchannel is composed of four groups of paratactic annuluses (Figure 1b). In each group, there are four rings formed by two semi-toroidal of different widths, with the wider one having a stagger angle of 120°. In addition, port numbers 1, 2, and 3 represent the entrances of three types of reagents, and port number 4 represents the exit of the product.

After assembly of the microchannel reactor, the precursor solution and other reagents were prepared. In the synthesis of CsPbBr_3_ QDs, PbBr_2_ and CsBr (0.085 g) were dissolved separately in a mixture of 10 mL DMF, 1 mL OA, and 0.5 mL OAm, to produce precursor solutions A (p-A) and B (p-B), respectively. Next, syringes containing p-A and p-B were set in syringe pump 1, and a syringe containing nonpolar solvent (toluene or chloroform) was set in syringe pump 2. The syringe pumps acted when the actual temperature reached the set value. After the collision between p-A and p-B, CsPbBr_3_–DMF solution was synthesized, and was then mixed with nonpolar solvent near entrance 3. Finally, the CsPbBr_3_ QDs, named QDs-M, passing the exit were collected.

For comparison between CsPbBr_3_ QDs by a microchannel reactor and those by a traditional synthetic method, we used a flask and mechanical stirring to produce QDs. First, the corresponding reagent ratio was calculated. Next, PbBr_2_ and CsBr were dissolved in 10 mL DMF. Then, OA and OAm were dropped in the DMF solution, under agitation, to obtain the precursor. Finally, the precursor was subjected to nonpolar solvent heating or cooling in a bath, and the synthetic process of CsPbBr_3_ QDs, called QDs-B, was completed.

### 2.3. Characterization

After synthesis of QDs-M, Ultraviolet visible (UV vis) absorption spectra were collected using a UV vis spectrometer (UV vis, Shimadzu, Kyoto, Japan) and emission spectra were measured using a fluorescence spectrophotometer (RF-6000, Shimadzu, Kyoto, Japan). Using an XRD (D8-Advance, Bruker, Karlsruhe, Germany) equipped with a Cu-Kα radiation source (λ = 0.15418 nm), the crystal structure was determined over the scanning angle (2θ) range from 5° to 80°. The morphology of QDs-M was characterized via a TEM (JEM-2100F, JEOL, Tokyo, Japan) operated at an accelerating voltage of 200 kV. The surface chemical element and valence states of QDs-M are analyzed by using XPS (Axis Ultra DLD, Kratos, Kyoto, Japan) equipped with an Al-Kα X-ray source. The QYs of QDs-M collected in exit of the reactor could be determined using the follow equation: [26,27,28]:

(1) $$Q_{x}{=Q}_{\mathrm{st}}\left(\frac{K_{x}}{K_{\mathrm{st}}}\right)\cdot \left(\frac{n_{x}^{2}}{n_{\mathrm{st}}^{2}}\right)$$

The absorption spectra and emission spectra of a reference material and of QDs-M were recorded over the same wavelength, so fitting straight lines appeared in the scatter diagram, with the absorbance as the *x*-axis, and the integral area of the emission spectrum as the *y*-axis. In Equation (1), *Q*, *K*, and *n* are QYs, the slope of that fitting straight lines, and the refractive index of the corresponding solution, respectively, where the subscript x represents the QDs-M sample, and the subscript st represents the reference material.

## 3. Results and Dicussion

### 3.1. The Structure and Morphology of CsPbBr_3_ QDs

**Figure 2:**
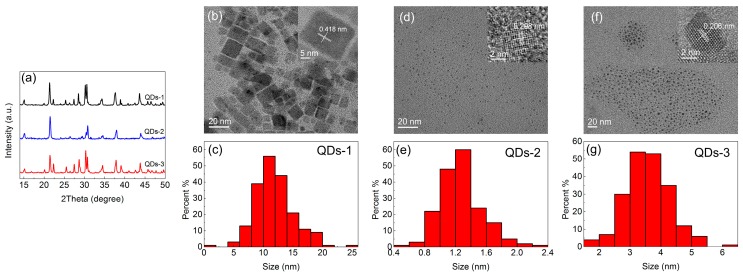
(**a**) XRD patterns of QDs-1, QDs-2, and QDs-3; (**b**) TEM image and (**c**) particle size statistical chart of QDs-1; (**d**) TEM image and (**e**) particle size statistical chart of QDs-2; (**f**) TEM image and (**g**) particle size statistical chart of QDs-3.

The XRD patterns and TEM images of three groups of QDs-M are shown in Figure 2. These QDs-M with different temperatures and velocities of the precursor solution are named as QDs-1, QDs-2, and QDs-3 (Table 1).

Based on the XRD patterns in Figure 2a, QDs-1 and QDs-3 are well coincident with PDF#18-0346 (a = b = 5.827 Å, c = 5.891 Å), and QDs-2 are in accordance with PDF#54-0751 (a = 9.843 Å, b = 4.127 Å, c = 5.891 Å). Each of the three patterns exhibits peaks at approximately 15°, 21.5°, 30.5°, 34.5°, 37°, and 44°, and the crystal indices to each peak are not the same. For 21.5°, QDs-1, QDs-2, and QDs-3 correspond to (110), (100), and (010), respectively.

**Figure 3:**
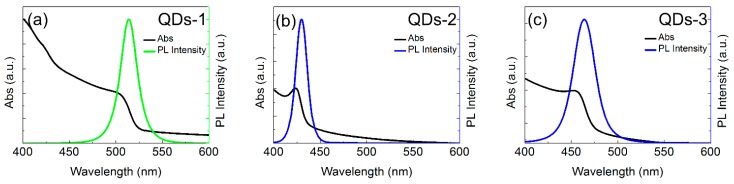
Absorption spectra and photoluminescence (PL) spectra of (**a**) QDs-1; (**b**) QDs-2; and (**c**) QDs-3.

As shown in Figure 3b,d,f, QDs-1 are dominated by a monoclinic morphology, whereas QDs-2 and QDs-3 prepared at different temperatures and velocities have no definite microstructure (orthorhombic, tetragonal, and spherical included). According to the HRTEM images shown at the top right corner, the crystalline interplanar spacings of these QDs-M are 0.418 nm, 0.208 nm, and 0.206 nm. The crystal indices of these HRTEM images are (110), (020), and (202), compared with similar interplanar spacings of 0.420 nm, 0.207 nm, and 0.207 nm obtained in XRD patterns. Low crystalline interplanar spacings also indicate the good crystallinity of QDs-M. Moreover, from the statistical charts of particle size (Figure 3c,e,g), the average sizes of QDs-1, QDs-2, and QDs-3 are 11.85 nm, 1.27 nm, and 3.89 nm, respectively, with sizes primarily in the range from 0.8 nm to 18 nm.

**Figure A1:**
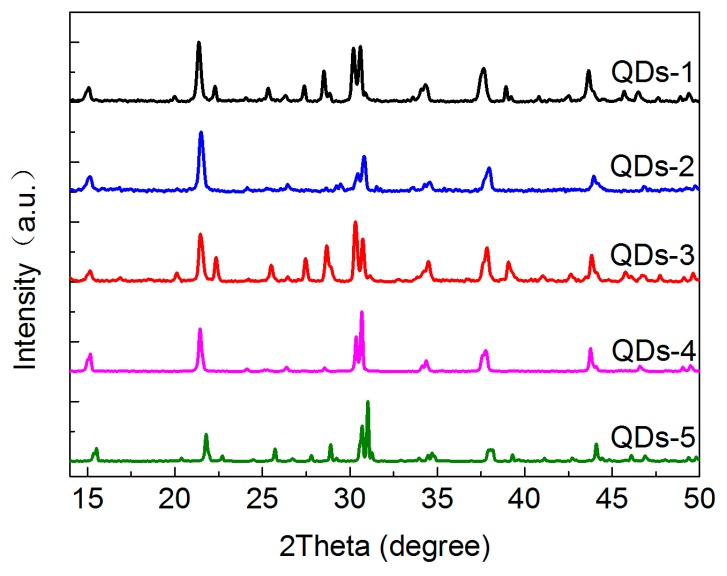
XRD patterns of QDs-1~QDs-5.

**Figure A2:**
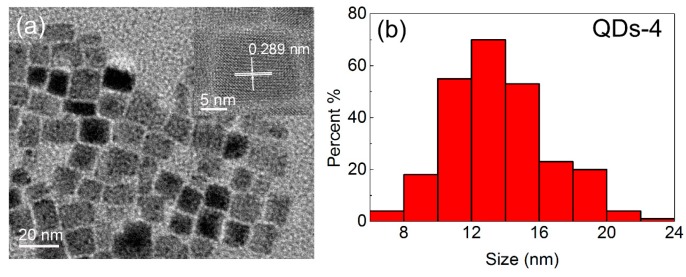
(**a**) TEM image and (**b**) particle size statistical chart of QDs-4.

**Figure A3:**
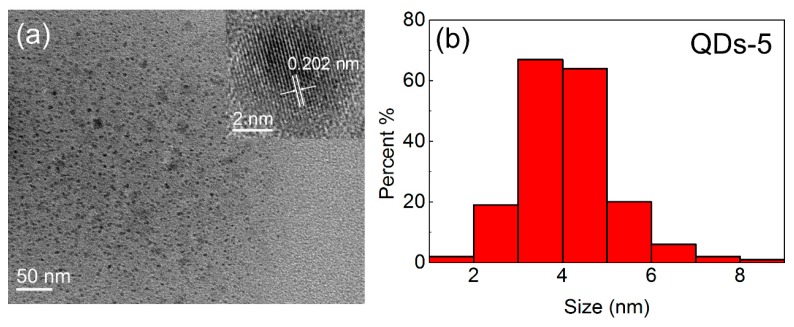
(**a**) TEM image and (**b**) particle size statistical chart of QDs-4.

In addition, two groups of QDs-M were synthesized with chloroform as weak polar solvents, which are named as QDs-4 and QDs-5 (Appendix A Table A1). The XRD patterns, TEM images, and particle size statistical charts of these samples are shown in Figure A1, Figure A2 and Figure A3. Comparing these five groups, a right shift occurs in the XRD patterns of QDs-M with the same weak polar solvent. The shorter the corresponding wavelength is, the more evident the right shift, in agreement with the effect of changing the halide [9,14]. The average sizes of QDs-4 and QDs-5 are 13.63 nm and 4.09 nm, respectively, which indicates that with chloroform, the structure of QDs-M can also be changed by different temperatures and velocities of the precursor solution.

### 3.2. Optical Properties of CsPbBr_3_ QDs

The absorption spectra and photoluminescence (PL) spectra of QDs-1, QDs-2, and QDs-3 are shown in Figure 3. All of them exhibit similar absorption transitions, but the excitonic absorption of QDs-2 is more obvious.

**Figure 4:**
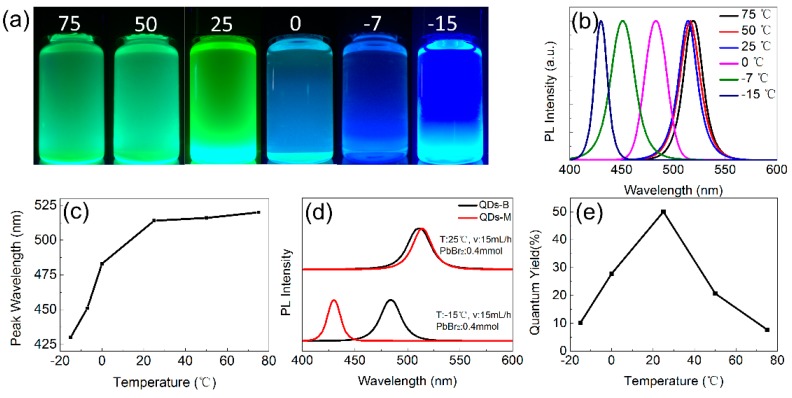
(**a**) Digital images under the UV light of 365 nm; (**b**) emission spectra; and (**c**) wavelength statistical chart of QDs-M at different reactor temperatures, where toluene is used as the weak polar solvent; (**d**) Comparison graph of the emission spectra of QDs-M and QDs-B with toluene as the weak polar solvent. (T represents temperature, v represents precursor flow velocity and PbBr_2_ represents dosage of PbBr_2_); (**e**) The QYs of QDs-M with different temperatures.

Furthermore, the wavelengths of QDs-M samples change with different reactor temperatures when using toluene as the weak polar solvent (Figure 4). An obvious blue-shift from 515 nm to 430 nm appears in the emission spectra with temperatures lower than 0 °C. Besides, a slight red-shift arises when reactor temperature is higher than 50 °C. Nevertheless, the synthesis of Br-QDs using the SR process at low temperature exhibited a little change in wavelength. The experimental error caused by so many uncontrollable factors resulted in the lack of a change in wavelength at different temperatures. In our work, temperature was applied to adjust the wavelength through precise control of both mass and heat in the microchannel. As shown in Figure 4d, in the comparison of the emission spectra of QDs-M and QDs-B synthesized at the same temperature, a wavelength shift of approximately 30 nm is observed in the QDs-B spectra. However, by altering the setting of the temperature controller, the scale of the QDs-M’s wavelength shift is 90 nm.

**Figure 5:**
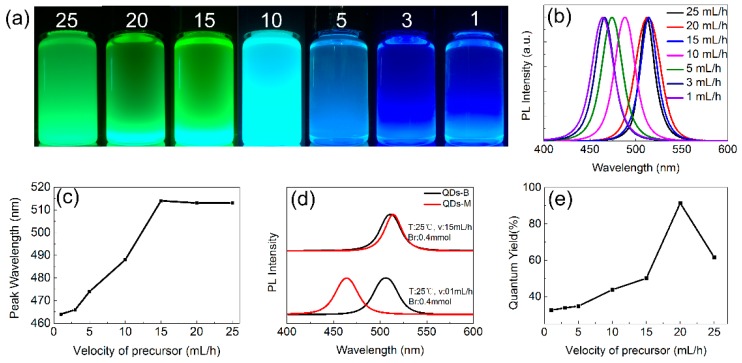
(**a**) Digital images under the UV light of 365 nm; (**b**) emission spectra; and (**c**) wavelength statistical chart of QDs-M at different velocities of the precursor solution, where toluene is used as the weak polar solvent; (**d**) Comparison graph of the emission spectra of QDs-M and QDs-B with toluene as the weak polar solvent. (T represents temperature, v represents precursor flow velocity and PbBr_2_ represents dosage of PbBr_2_); (**e**) The QYs of QDs-M with different velocities.

The PL emission of QDs-M also changes with different velocities of the precursor solution (Figure 5). An obvious blue-shift from 515 nm to 464 nm appears with velocity lower than 5 mL/h. When a small amount of precursor solution was dropped into the toluene in SR method, the PL emission of CsPbBr_3_ QDs has little change (Figure 5d). The precise mixture of small amount of liquid is realized by a microchannel reactor, which is fairly difficult for a conventional flask. As shown in Figure 2, the shorter wavelength matches the smaller sizes, proving that the spectral shift can be attained by different size via temperature-control and velocity-control.

**Figure A4:**
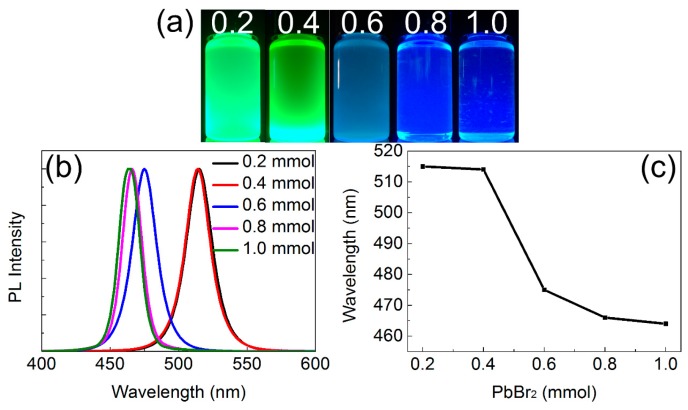
(**a**) Digital images under the UV light of 365 nm; (**b**) emission spectra; and (**c**) wavelength statistical chart of QDs-M with different dosage of PbBr_2_, where toluene is used as nonpolar solvent.

**Figure A5:**
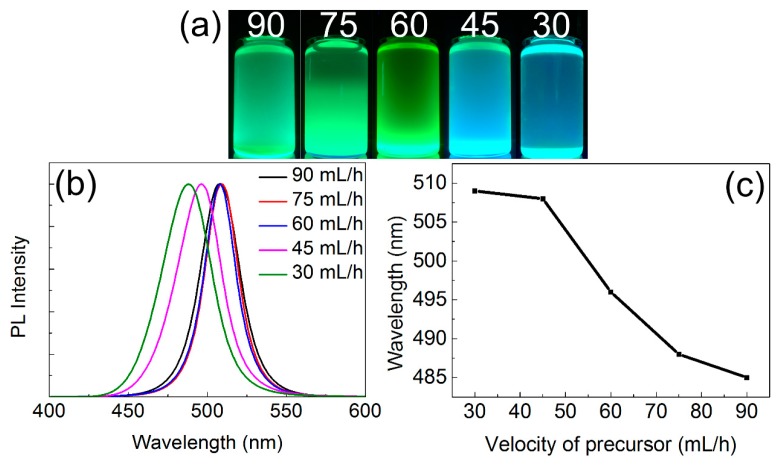
(**a**) Digital images under the UV light of 365 nm; (**b**) emission spectra; and (**c**) wavelength statistical chart of QDs-M with different velocity of precursor, where chloroform is used as nonpolar solvent.

**Figure A6:**
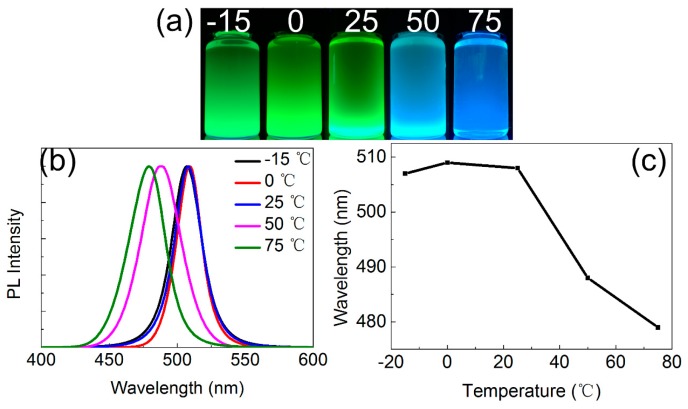
(**a**) Digital images under the UV light of 365 nm; (**b**) emission spectra; and (**c**) wavelength statistical chart of QDs-M with different reaction temperature, where chloroform is used as nonpolar solvent.

**Figure A7:**
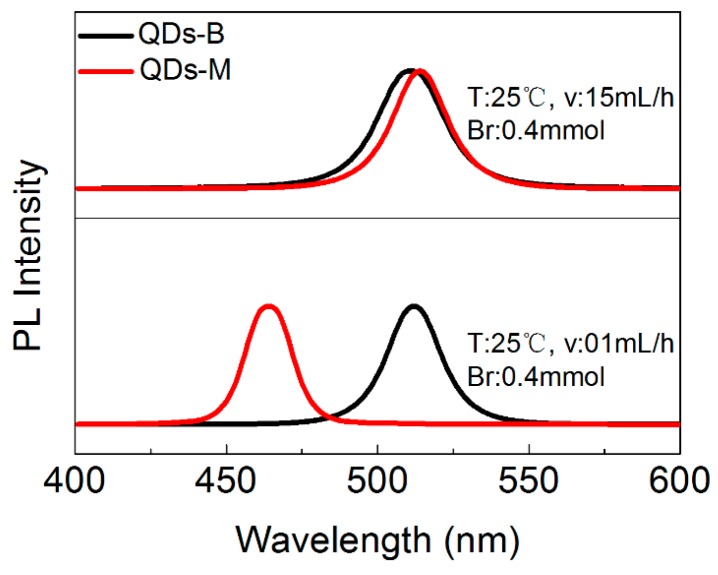
Comparison graph of QDs-M’s and QDs-B’s emission spectrum with toluene as nonpolar solvent as nonpolar solvent. (T represents temperature, v represents precursor flow velocity and Br represents dosage of PbBr_2_.)

**Figure A8:**
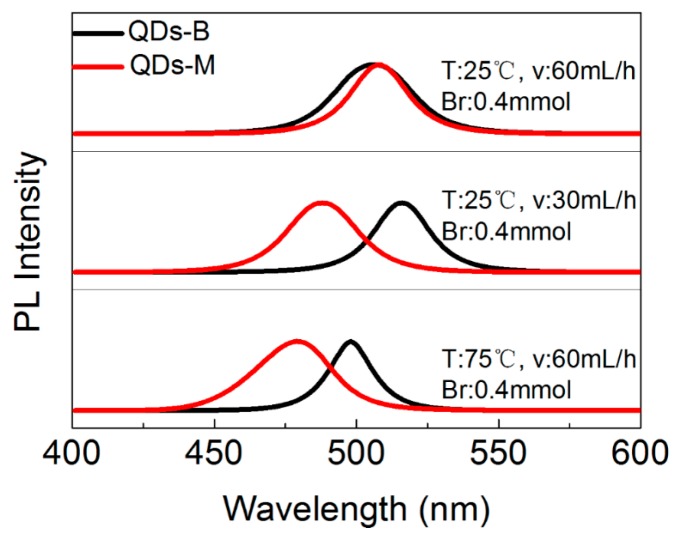
Comparison graph of QDs-M’s and QDs-B’s emission spectrum with toluene as nonpolar solvent as nonpolar solvent. (T represents temperature, v represents precursor flow velocity and Br represents dosage of PbBr_2_.)

Similar blue-shifts arise for different dosages of PbBr_2_ with toluene, or for different velocities and different reaction temperatures with chloroform. These blue-shifts are from 515 nm to 466 nm if the dosage of PbBr_2_ is more than 0.6 mmol (Figure A4); similarly, the shifts are from 509 nm to 488 nm, or from 509 nm to 479 nm if the precursor velocity is lower than 45 mL/h or the temperature is higher than 50 °C, separately, when using chloroform (Figure A5, Figure A6, Figure A7 and Figure A8).

The broad range of PL emission shift of QDs-M is primarily achieved via three advantages of the microchannel reactor. The first advantage is the exact control of the reagent in it. Secondly, a microchannel has superior heat transfer performance because of the ultrahigh specific surface area and a better mixing effect, resulting from the rational design of the microchannel geometry. Finally, the precursor of QDs-M is in contact with the weak polar solvent in a proportional manner at any time, different from a little precursor in contact with the large amount of weak polar solvent instantaneously in the growth of QDs-B. The synthesis of QDs-M has a much shorter reaction time without inert gas protection; it is also capable of achieving a broad spectrum without changing the halide, by control the syringe pump and the temperature controller.

**Figure A9:**
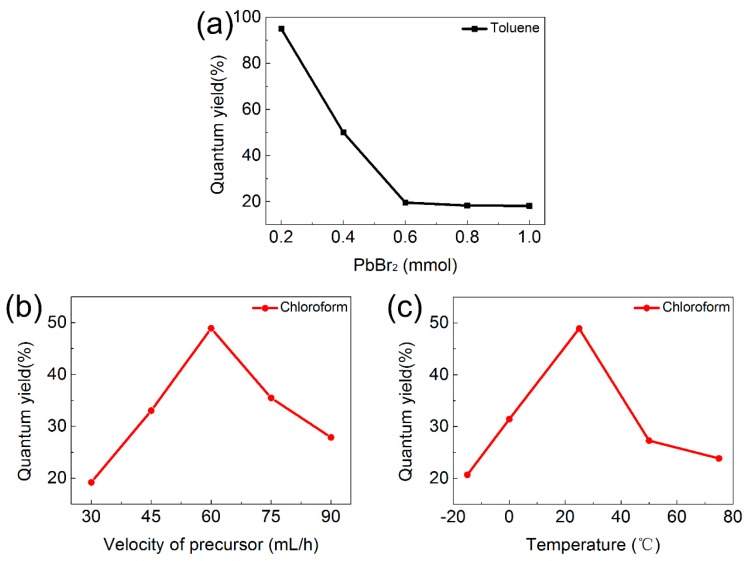
QY image of QDs-M by (**a**) changing dosage of PbBr_2_, (**b**) changing precursor flow velocity with toluene as nonpolar solvent, (**c**) changing precursor flow velocity with chloroform as nonpolar solvent.

For toluene, the highest QYs of QDs-M appears at 25 °C with temperature changed, or at 20 mL/h with different velocities, as shown in Figure 4e and Figure 5e. With higher temperature, the QYs drop for the exciton binding energy reduced. Combined with the emission shift, the QYs of green QDs-M was found to be higher than that of the blue ones. The QDs-M, via a microchannel, can reach upper QYs without intentional surface passivation, for green CsPbBr_3_ QDs that have been reported [7,10]. From the QYs of other groups (Figure A9), QDs-M with toluene have QYs values up to 94%, generally higher than those with chloroform.

**Figure 6:**
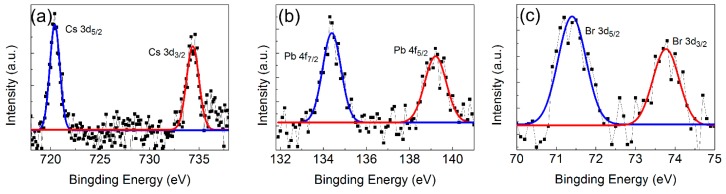
High resolution XPS analyses of QDs-M corresponding to (**a**) Cs 3d; (**b**) Pb 4f; and (**c**) Br 3d.

Moreover, the surface chemical element and valence states of QDs-M are analyzed by XPS (Figure 6a–c). From the high resolution XPS spectra of Cs, Pb, and Br atoms, the binding energy curves of Cs 3d_3/2_ and Cs 3d_5/2_ appear at 734.4 eV and 720.4 eV, respectively. In addition, the peaks of Pb 4f_5/2_ and Pb 4f_7/2_ are located at 139.2 eV and 134.5 eV. Besides, the weak intensity profile of Br 3d_3/2_ and Br 3d_5/2_ are situated at 73.8 eV and 71.4 eV, respectively.

### 3.3. The PL Spectral Shift Mechanism of CsPbBr_3_ QDs’ Changeable Wavelength

The quantum confinement effect indicates that the wavelength of QDs can change with variation of the band gap due to the nanocrystal size [29,30]. In the HI method, the temperature of the water bath is capable of influencing the peaks of the CsPbBr_3_ QDs samples in the emission spectra, with a smaller crystal size corresponding to a blueshift of the wavelength; however, no further discussion was provided on the effect of temperature [31]. Following that report, we introduce the parameters of not only temperature, but also reagent dosage and precursor velocity, and discuss the impact of these parameters on crystal size during crystallization to further explain the wavelength change of QDs-M.

**Figure 7:**
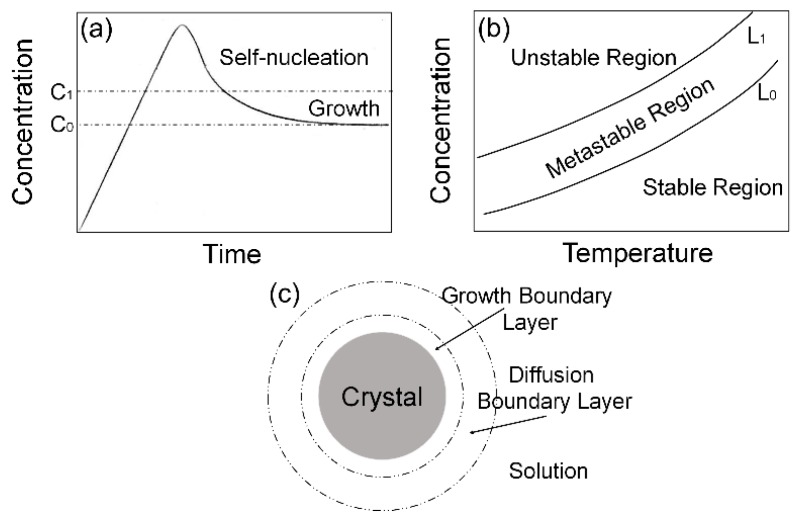
(**a**) Schematic diagram of the three stages of crystallization; (**b**) schematic diagram of the supersaturation curve and the supersolubility curve; (**c**) schematic diagram of the two boundary layer in the crystal growth stage.

As a type of nanocrystal, CsPbBr_3_ QDs’ crystallization includes three stages: the induction period, nucleation stage and crystal growth stage. In the induction period, the solution reaches a relatively stable state instead of nucleating immediately, when the solubility corresponding to the present temperature and pressure is exceeded. If the amount of solute is sufficient, then the energy barrier is exceeded with propulsion of supersaturation, and the liquid–solid interface appears. The accompanying spontaneous nucleation in this process indicates that the nucleation stage is attained, in which the solute concentration decreases [32]. When solute concentration decreases to the lowest supersaturation required for nucleation, the solute particles accumulate on the crystal nucleus formed in the prior nucleation stage, and then, the crystal grows under the low supersaturation, approximating to the stable state, which is called the crystal growth stage. As shown in Figure 7a, in the case that the saturation is too high, the solute tends to nucleate spontaneously, rather than crystal growing in this stage, because low saturation is a prerequisite [33]. Occurring simultaneously in the crystal growth stage is the Ostwald ripening process [34,35], presenting the melting of small crystal grains as well as the growth of a large crystal grain. Because of the higher surface energy of the small particles, a decrease of the small particles reduces the free energy to stabilize the whole solution, with the solute concentration increasing and the crystal growth process promoted. As the solubility of the solute declines to C_0_, there is no supersaturation as the driving force, and the whole crystallization process ends.

The latter two stages, namely, the nucleation stage and the crystal growth stage, determine the final crystal size; thus, the following discussion focuses on various factors affecting the crystal size in these two stages. A large number of tests confirmed that the relationship between supersaturation and crystallization is as shown in Figure 7b, with an example of a substance whose solubility increases as the temperature rises. In Figure 7b, L_0_ represents the solubility curve and L_1_ represents the concentration curve, where the solution is supersaturated, and nucleates spontaneously. That concentration-temperature diagram is divided into three regions by L_0_ and L_1_: the stable region, metastable region, and unstable region. The stable region below L_0_ is the portion where the solution cannot nucleate because of unsaturation, whereas initiative nucleus formation occurs in the unstable region above L_1_. Relatively, the area between L_0_ and L_1_ is a metastable region in which no nucleation occurs, but a pre-existing crystal nucleus grows via supersaturation propelling. This region, proposed by Ostwald in 1893 and developed into a mature theory by the research of Miers, Young, etc. [36], accords with C_0_~C_1_ (Figure 7a).

According to the heterogeneous nucleation theory developed by Hirth, Pound, Walton, and Zinsmeister [37,38], the primary factors affecting the nucleation rate includes the degree of supersaturation, temperature, and pressure, with the influence of supersaturation being much greater than the others. Note that the location of L_1_ changes in a certain scale, affected by parameters such as availability of stirring and the stirring intensity. Because of the violent mixing via the microchannel, movement of L_1_ is low enough to assume that L_1_ remains at the same position. Consequently, within all the reaction parameters, being deeper in the metastable region brings a higher degree of supersaturation, resulting in a higher nucleation rate and more crystal nuclei, as shown in Figure 7b. If the number of crystal nuclei increases due to high supersaturation during the nucleation stage, then the amount of solute accumulating around every crystal nucleus will diminish, thereby reducing the crystal grain size overall.

As early as 1925, the von Weimarn rules proposed by Weimarn also described the relationship between crystal size and supersaturation during the period of crystallization [39]. One of the rules is that a lower degree of supersaturation during the induction period leads to a higher crystal dimension. Subsequently, many researchers meliorated and supplemented the von Weimarn rules; for example, Jame K. Baird and co-workers deduced a calculation formula [40]:

(2) $${\langle J\rangle }_{P}\propto \sigma_{0}^{\left(2-P\right)/4}$$

where ${\langle J\rangle }_{p}$ represents the grain size, $\sigma_{0}^{}$ represents the initial degree of supersaturation and is a dimensionless parameter, and *P* ≥ 2, in general. This formula also proves that the higher the degree of supersaturation, the smaller of the crystal is.

According to the solubility rule, i.e., “like dissolves like”, similar polarities bring higher intersolubility. After CsPbBr_3_–DMF solution is added to the weak polar solvent, the crystals are precipitated via extremely high supersaturation, as a result of the sharp decline of solubility origination from the large difference in polarity between CsPbBr_3_ and DMF–weak polar solvent mixed solution. Supersaturation during crystallization of CsPbBr_3_ QDs is related to the reaction temperature, the types of weak polar solvent, and the ratio of CsPbBr_3_–DMF solution and weak polar solvent, so supersaturation can change via adjustment of these three parameters to attain CsPbBr_3_ QDs with different sizes.

Firstly, an interesting fact is that the QDs-M’s wavelength decreases with temperature reduction when using toluene, whereas, adopting chloroform, the QDs-M’s wavelength decreases with temperature rise. The main reason is that the solubility of toluene in the polar solvent increases with rising temperature, whereas the while solubility of chloroform decreases. Taking water as an example, 0.035 g toluene or 1.062 g chloroform can be dissolved in 100 g water at 0 °C, and 0.057 g toluene or 0.776 g chloroform can be dissolved in 100 g water at 30 °C; the reduction in solubility corresponds to an increase in supersaturation.

Secondly, as shown in Figure 5a,b and Figure A5a,b, the reduction of precursor flow velocity means that the ratio of CsPbBr_3_ to the weak polar solvent decreases, resulting in increased supersaturation, diminished crystal size, and spectral blue shift.

Finally, the crystal growth stage is primarily divided into two parts, transport of solute from solution to the crystal interface, and sedimentation of that solute on the interface included (Figure 7c). The transport occurs in zones between homogeneous solution and the diffusion boundary layer, and the diffusion rate depends on the convection and diffusion conditions [41]. In addition, the sedimentation occurs in the zone between the growth boundary layer and the crystal interface. According to the growth model for helix growth and inference of Chernov, etc., there is a positive correlation relation between deposition rate *R* and degree of supersaturation σ [42,43,44]:

(3) R = kσ

where *k* is the kinetics coefficient of the effective growth. In Figure A4a,b, the dosage of PbBr_2_ is changed moderately, and the QDs-M production remains unchanged, so original supersaturation stays the same. If the ratio of PbBr_2_ QY and CsBr is greater than two, then the reducing rate of crystal growth, due to excess PbBr_2_ impeding the diffusion of solute, leads to the smaller grain size of QDs and blueshift in the emission spectrum.

### 3.4. The Application of CsPbBr_3_ QDs in LEDs

**Figure 8:**
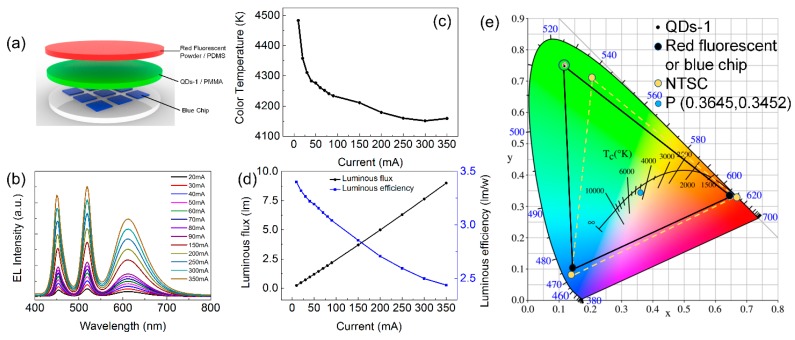
(**a**) Diagram of the LED device combining red fluorescent powder/PDMS composites, QDs-1/PMMA mixture with blue chips; (**b**) EL spectrum of the LED device at different currents; (**c**) color temperature of the LED device at different currents; (**d**) luminous flux and luminous efficiency of the LED device at different currents; (**e**) CIE color coordinates of QDs-1 at different currents and NTSC standard.

For application of QDs-M, a thin film was fabricated by mixing QDs-1 and polymethyl methacrylate (PMMA). An LED device was assembled by red fluorescent powder/polydimethylsiloxane (PDMS) composites, the QDs-1/PMMA mixture, and blue LED chips (Figure 8a). Next, the electroluminescence (EL) spectrum, color temperature, luminous flux, and luminous efficiency of this LED device at different currents were attained (Figure 8b–d). As the current increased, the color temperature of this LED device reduced from 4500 K to 4100 K, luminous efficiency was reduced, but the luminous flux gradually increased. It can also be seen that the CIE color coordinates marked as P (0.3645, 0.3452) in Figure 8e, so warm white light is generated. Besides, the CIE color coordinates of QDs-1 present a pure green color. In addition, the LED device covers an area that is much larger than that of conventional LEDs, reaching 112% NTSC. These results indicate that QDs-M produced by a microchannel reactor can be applied in an LED display.

## 4. Conclusions

In summary, we found that the PL emission of QDs can be controlled effectively via the process parameters such as reaction temperature and velocity of the precursor solution. Therefore, we designed an ingenious temperature controlled microchannel reactor with a dislocated snake-like shape. Though adjusting the temperature, the precursor flow velocity, and the dosage of PbBr_2_, QDs-M by a microchannel reactor have a fairly wide spectral range from 520 nm to 430 nm, more than that of QDs by a conventional flask, without the production of inert gases and the adjustment of reagents. Due to the reagent proportional contact and homogeneous heating or cooling at any time in the microchannel, the supersaturation was simply changed by setting of the syringe pump and the temperature controller. Consequently, the final crystal grain size and the PL emission were changed. Our synthesis of QDs-M with high crystallinity and with QYs of more than 90%, had the advantages of a fast reaction time, a wide spectrum range, and precise control; thus, our synthesis approach was expected to promote the application of CsPbBr_3_ QDs in LED backlight unit, solar cells, and lasers.

## Figures and Tables

**Table 1:** Parameters of three groups of QDs-M, weak polar solvent, dosage of PbBr_2_, precursor flow velocity, temperature, and wavelength.

Group	Weak Polar Solvent	PbBr_2_ (mmol)	Precursor Flow Velocity (mL/h)	Temperature (°C)	Wavelength (nm)
QDs-1	Toluene	0.4	15	25	515
QDs-2	Toluene	0.4	15	−15	430
QDs-3	Toluene	0.4	1	25	464

## Appendix A

**Table A1:** Parameters of five groups of QDs-M, nonpolar solvent, dosage of PbBr_2_, precursor flow velocity, temperature, and wavelength.

Group	Nonpolar Solvent	PbBr_2_ (mmol)	Precursor Flow Velocity (mL/h)	Temperature (°C)	Wavelength (nm)
QDs-1	Toluene	0.4	15	25	515
QDs-2	Toluene	0.4	15	−15	430
QDs-3	Toluene	0.4	1	25	464
QDs-4	Chloroform	0.4	60	25	508
QDs-5	Chloroform	0.4	60	75	479
